# Supplementary material for: Cryo-EM structures of plant Augmin reveal coiled-coil assembly, antiparallel dimerization, and NEDD1 binding
Source: Nat Commun. 2025 Dec 12;16:11440. doi: 10.1038/s41467-025-66332-4 (PMC12748588; doi:10.1038/s41467-025-66332-4)
Supplement: Supplementary file 1 — Supplementary Information [file 41467_2025_66332_MOESM1_ESM.pdf]

Cryo-EM structures of plant Augmin reveal coiled-coil assembly, antiparallel dimerization, and NEDD1 binding

Md Ashaduzzaman*, Aryan Taheri*, Yuh-Ru Julie Lee , Yuqi Tang, Shubham Mittal, Fei Guo, Faruck Morcos, Stephen D. Fried, Bo Liu, Jawdat Al-Bassam

Supplementary Figures 1-16 and Supplementary Tables 1-5

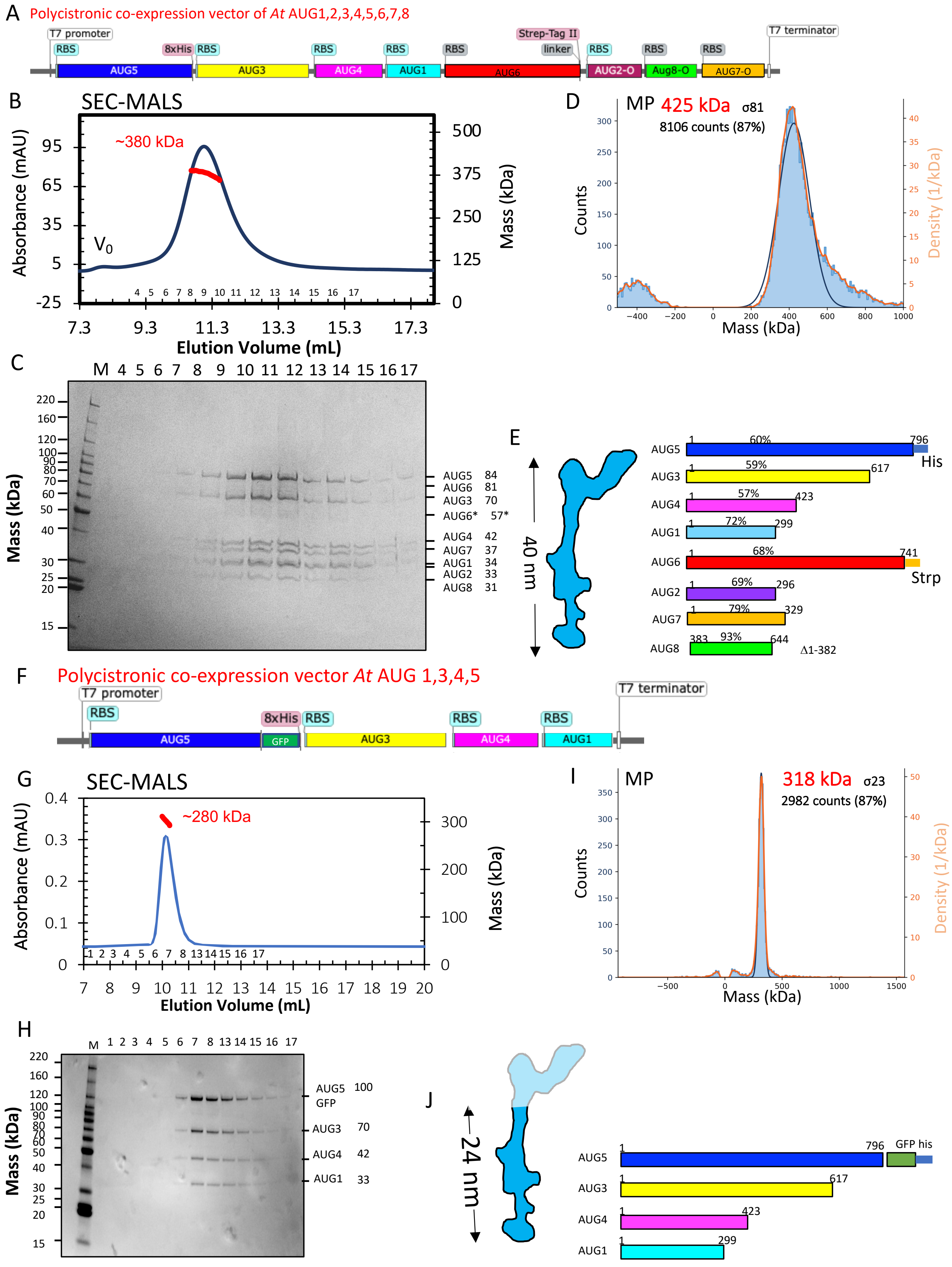

Supplementary Fig. 1: **Biochemical reconstitution and characterization of *Arabidopsis thaliana***

Augmin.

A) Organization of the AUG1,2,3,4,5,6,7,8 polycistronic vector for expressing assemblies in bacteria showing the T7 promoter, ribosomal binding site (RBS) and open reading frame (ORF) order for each of the eight subunits in consistent color coding shown for structures.

B) Size exclusion chromatography (SEC) with multi-angle light scattering (SEC-MALS) for AUG1,2,3,4,5,6,7,8 hetero-octameric assemblies revealing their overall mass to be 380 kDa (n=1).

C) SDS-PAGE of SEC-fractions revealing the eight AUG subunits of traces shown in B. AUG6* is degradation product of AUG6. M noted lane is standard and each fractions shown in (B) was ran on gel.

D) Mass photometry (MP) measured for masses purified AUG1,2,3,4,5,6,7,8 fitted with Gaussian distribution revealing masses of 425 KDa (n=>5).

E) Left the overall organization of the 40 nm hetero-octameric full Augmin particle, Right, linear scheme for all *AT* AUG subunits in the assembly as shown in Figure 1 in consistent color coding. Percentage values above indicate coverage regions of polypeptide in mass spectrometry of purified assemblies.

F) Organization of the AUG1,3,4,5 polycistronic vector for expressing assemblies in bacteria showing the T7 promoter, ribosomal binding site (RBS) and open reading frame (ORF) order for each of the four subunits with AUG5-containing a C-terminal Green Fluorescent Protein (GFP) and His tag.

G) Size exclusion chromatography with multi-angle light scattering (SEC-MALS) for hetero-tetrameric (AUG1,3,4,5) Augmin assemblies revealing their overall mass to be 280 kDa (n=1).

H) SDS-PAGE of SEC-fractions revealing the four AUG subunits of traces shown in G. M noted lane is standard and each fractions shown in (G) was ran on gel.

I) Mass photometry measured for masses purified AUG1,3,4,5 assemblies fitted with Gaussian distribution revealing masses of 318 KDa (n=>3).

J) Left the overall organization of the 24 nm hetero-tetrameric Augmin particle, Right, linear scheme for all *AT* AUG subunits in the assembly as shown in Figure 1.

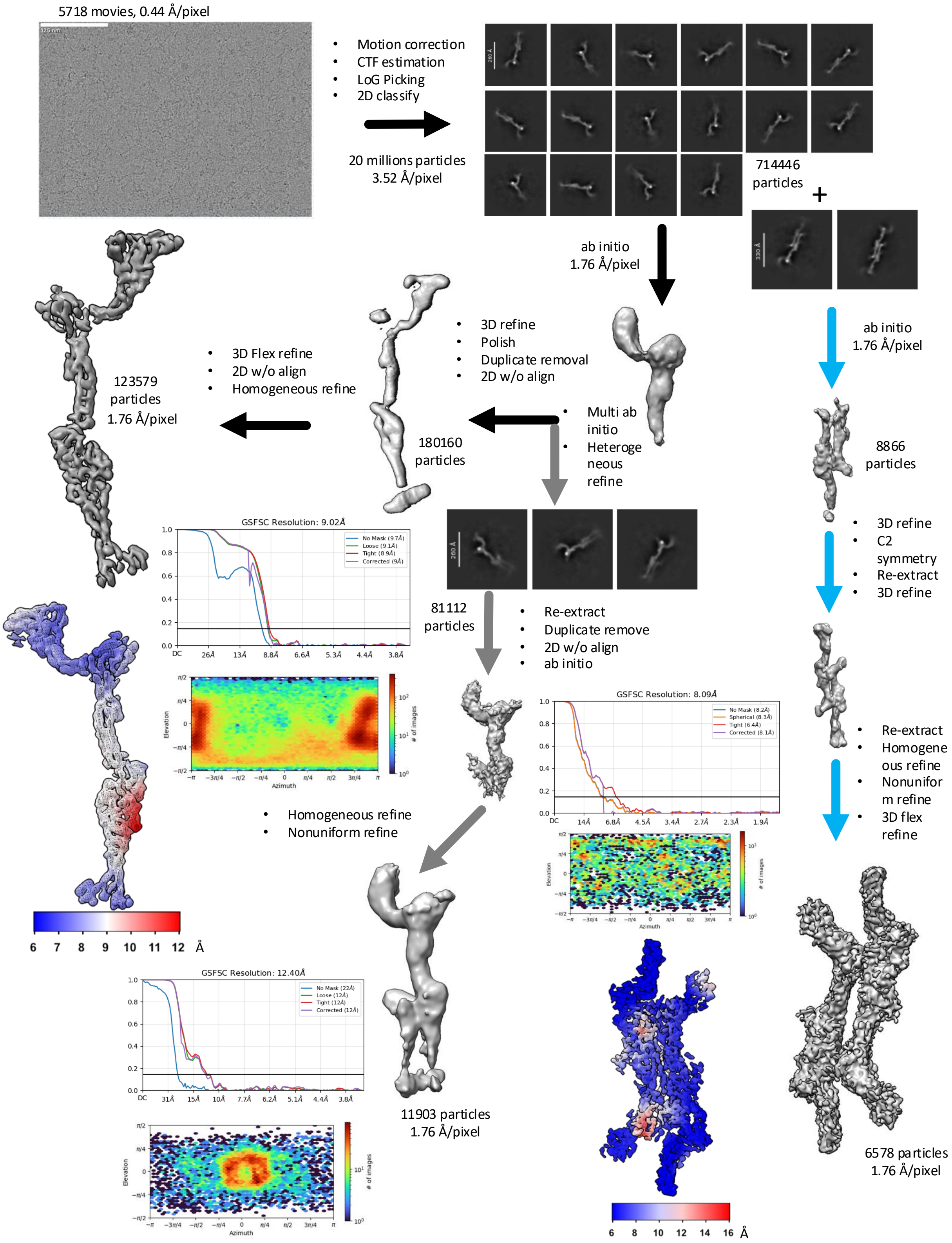

Supplementary Fig. 2: **Cryo-EM processing scheme for the full hetero-octameric (AUG1,2,3,4,5,6,7,8) Augmin leading to 10 Å cryo-EM structure.**

Top to bottom, hetero-octameric (AUG 1,2,3,4,5,6,7,8) Augmin cryo-EM dataset (example image shown on left; scale bar is 125 nm) was collected, and raw images were pre-processed using motioncorr2, CTFFind3 then used pick and identify coordinates for particle images, which were processed using a combination of RELION 3.0-4.0 and CryoSPARC 3.0-4.1. Multiple cycles of 2D classification identified three types of assemblies: top left 2D class averages, full Augmin assemblies, center middle, 2D-class averages, Augmin with additional extended regions (Augmin 1.5), and left 2D class averages, Augmin C2 symmetric dimers. Cycles of auto-3D refinement, flex-refine polishing, homogeneous refine in CryoSPARC 3.1 led to three reconstructions. These reconstructions are shown with their CryoSPARC angular distributions, Fourier shell correlation (FSC) curves and local resolution colored map. Center left, 10 Å-reconstruction of full Augmin assembly. Lower center, a 12 Å reconstruction of Augmin 1.5 with additional extended domain. Lower right, 12 Å C2 Augmin dimer reconstruction is shown. Final processing statistics are described in Table I.

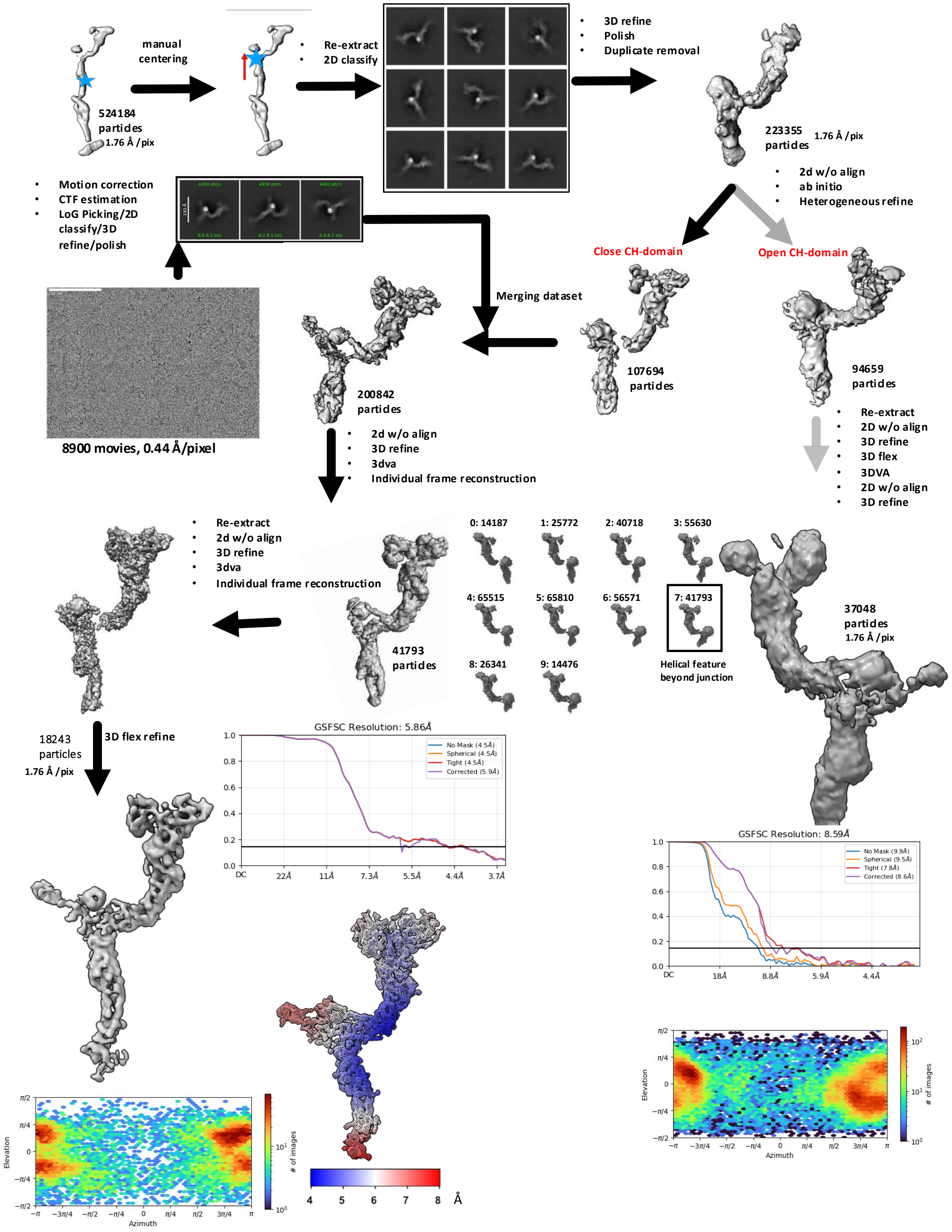

Supplementary Fig. 3: **Cryo-EM processing scheme for the hetero-octameric (AUG1,2,3,4,5,6,7,8) Augmin V-junction stem to 7.3 to 12 Å cryo-EM structure in the open and**

closed state.

Top to bottom, the hetero-octameric (AUG 1,2,3,4,5,6,7,8) V-junction stem particles in Supplementary Fig. 2 were re-centered and reextracted as shown in top left centering around the V-junction steam region, leading to refined class averages shown on top center. A second cryo-EM dataset for AUG1,2,3,4,5,6,7,8 assemblies (example image shown on left; scale bar is 125 nm) was collected, and raw images were pre-processed using motioncor2, CTFFind3 then used pick and identify coordinates for particle images, which were processed using a CryoSPARC 4 leading to 2D class averages shown middle left center. Multiple cycles of 3D refinement, heterogenous refine identified two types of V-junction stem assemblies: closed dual CH-dimer Head domain Left, open dual CH-dimer head domain. Particles from both datasets were merged for each of these two. Center middle, 3D classification led to a single class with improved features for the closed state. The dual CH-dimer closed state particles were re-extracted at smaller pixel size 3D auto-refined, 3D-variability refined (3DVA) then flex refined leading to a reconstruction at 7 Å resolution. Particles for the open state were auto-refined and leading to a 10 Å reconstruction of the Augmin V-junction-stem at in the open CH-dimer state.

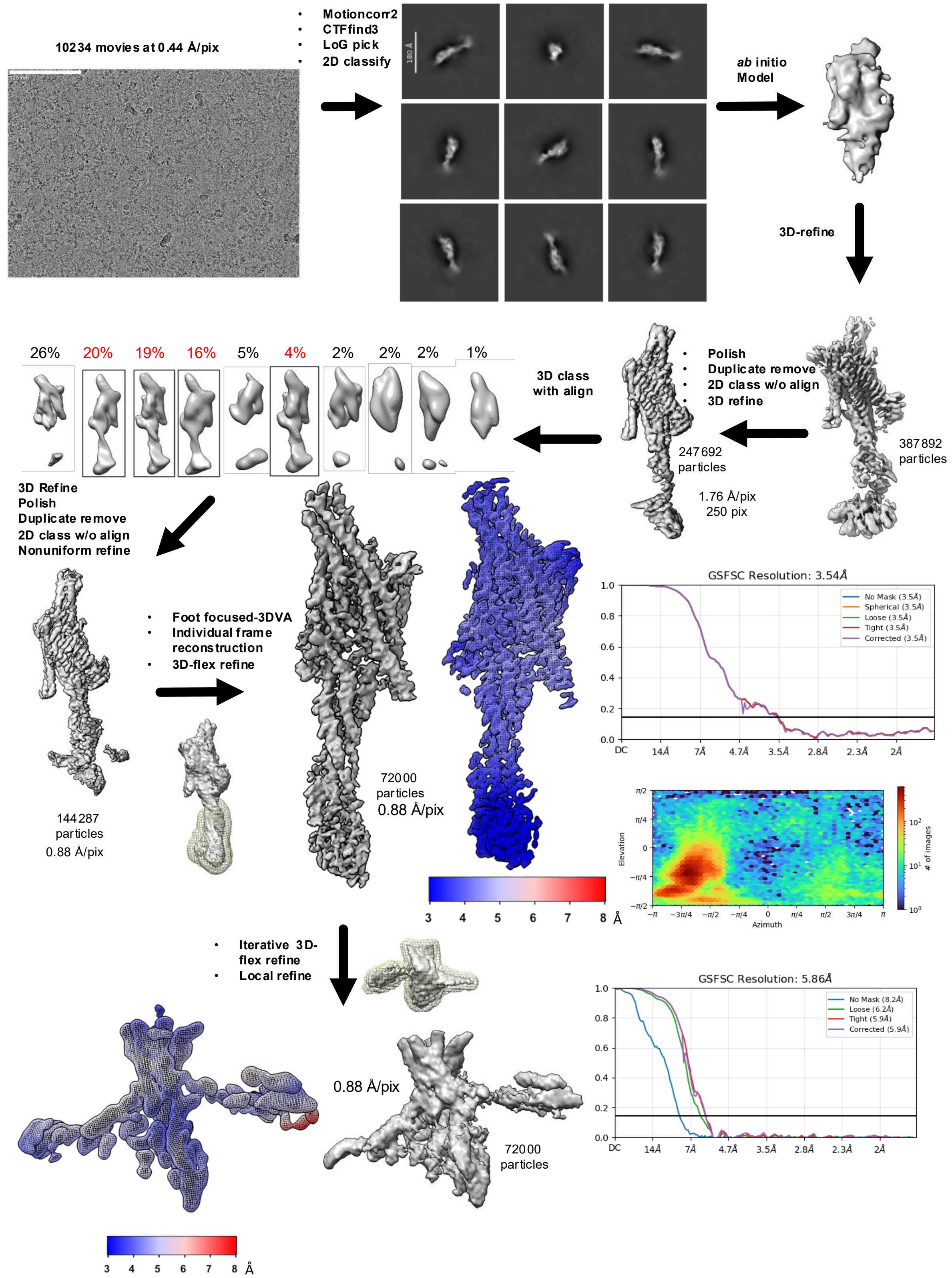

Supplementary Fig. 4: Cryo-EM data processing scheme for the hetero-tetrameric (AUG1,3,4,5) Augmin leading to a 3.7-6.0 A resolution map for the extended region

Top to bottom, cryo-EM data movies for hetero-tetrameric (AUG 1,3,4,5) Augmin particles (example image on top left, scale bar is 125 nm) was collected then pre-processed, using motioncorr2, CTFFind3 then used pick and identify coordinates for particle images, as described, leading to 2D-class averages shown on top center. Class averages were used to generate ab initio model top right, which was refined and then further polished and duplicate removed. This is followed by 3D classification with alignment leading to four classes which were combined, then followed the step shown in center left leading to a refined structure at 3.7 Å resolution for the extended region showing clear helical and side chain density shown in center. The lower section of the extended region was 3D-flex refined leading to a 6 Å structure for the tripod region.

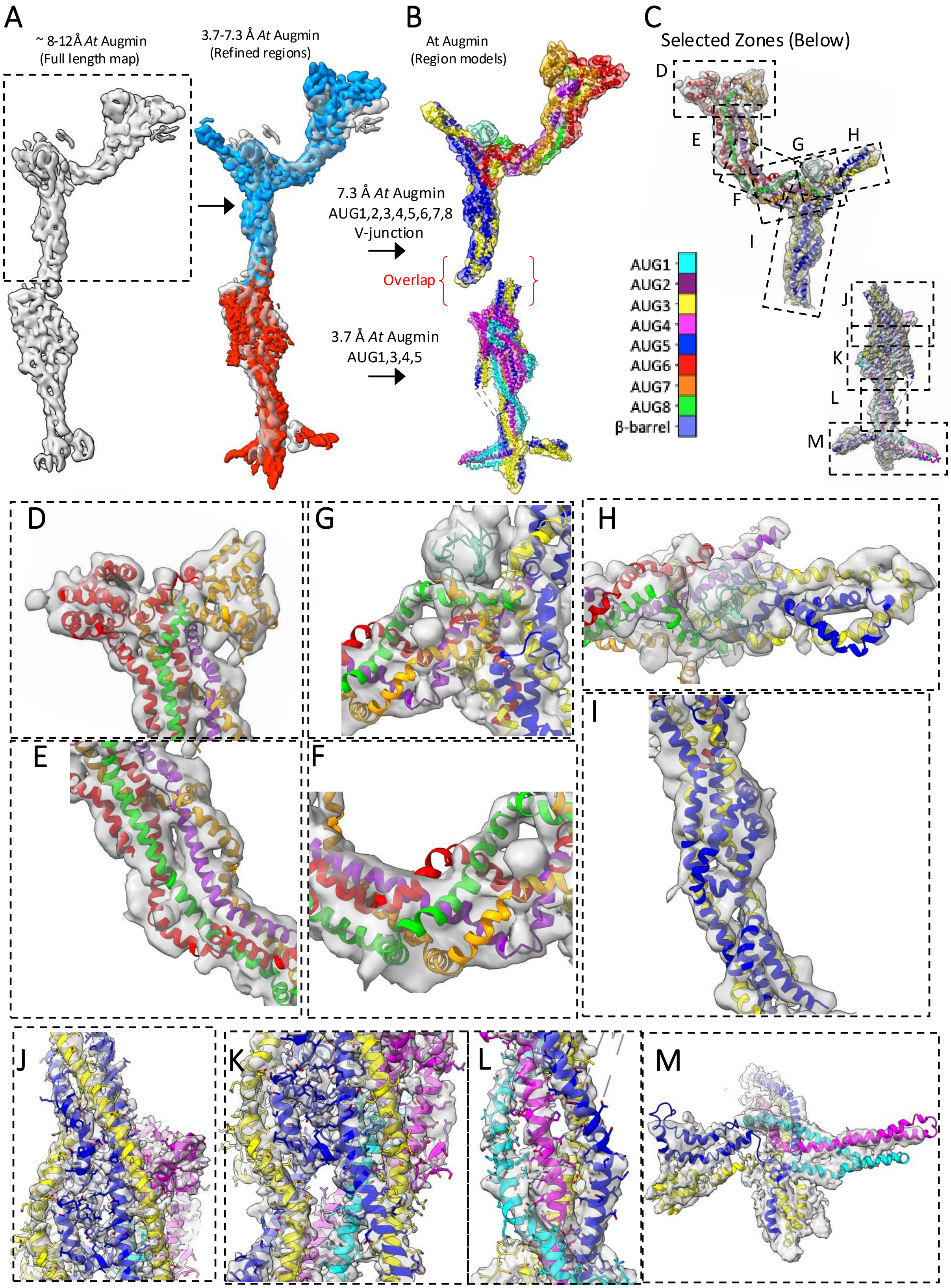

Supplementary Fig. 5: **Composite maps for a full *de novo* Augmin hetero-octamer model**

A) Right, 10 Å Cryo-EM map for Full Augmin hetero-octamer (AUG1,2,3,4,5,6,7,8) generated as presented in Supplementary Fig. 2. Left, aligned cryo-EM map (transparent white) as shown on left, overlaid with 7.3 Å map for the V-junction stem (blue) generated as described in Supplementary Fig. 3, and the 3.7 Å AUG1,3,4,5 Augmin extended region map generated as described in Supplementary Fig. 4

B) Segmented V-junction-stem map fitted with modeled AUG1,2,3,4,5,7,8 regions is shown on the top, with the head region of AUG6,7 CH-domain dimer in the closed state. The extended region map is shown on the bottom. The overlap region composed of four helices from AUG3,5 is marked by red parentheses. Subunit color guide shown in on right.

C) A Guide to close up views of the modeled regions shown in B (rotated by 180° from C). The V-junction and stem map zones marked with D-I boxes, representing different regions. The extended region is shown below with zones marked with J-M boxes representing different regions

D-I) Close-up views for different regions of V-junction stem model to map fits with guide to subunit colors shown in B

J-M) Close-up views for different regions of the extended region model to map fits with guide to subunit colors shown in B.

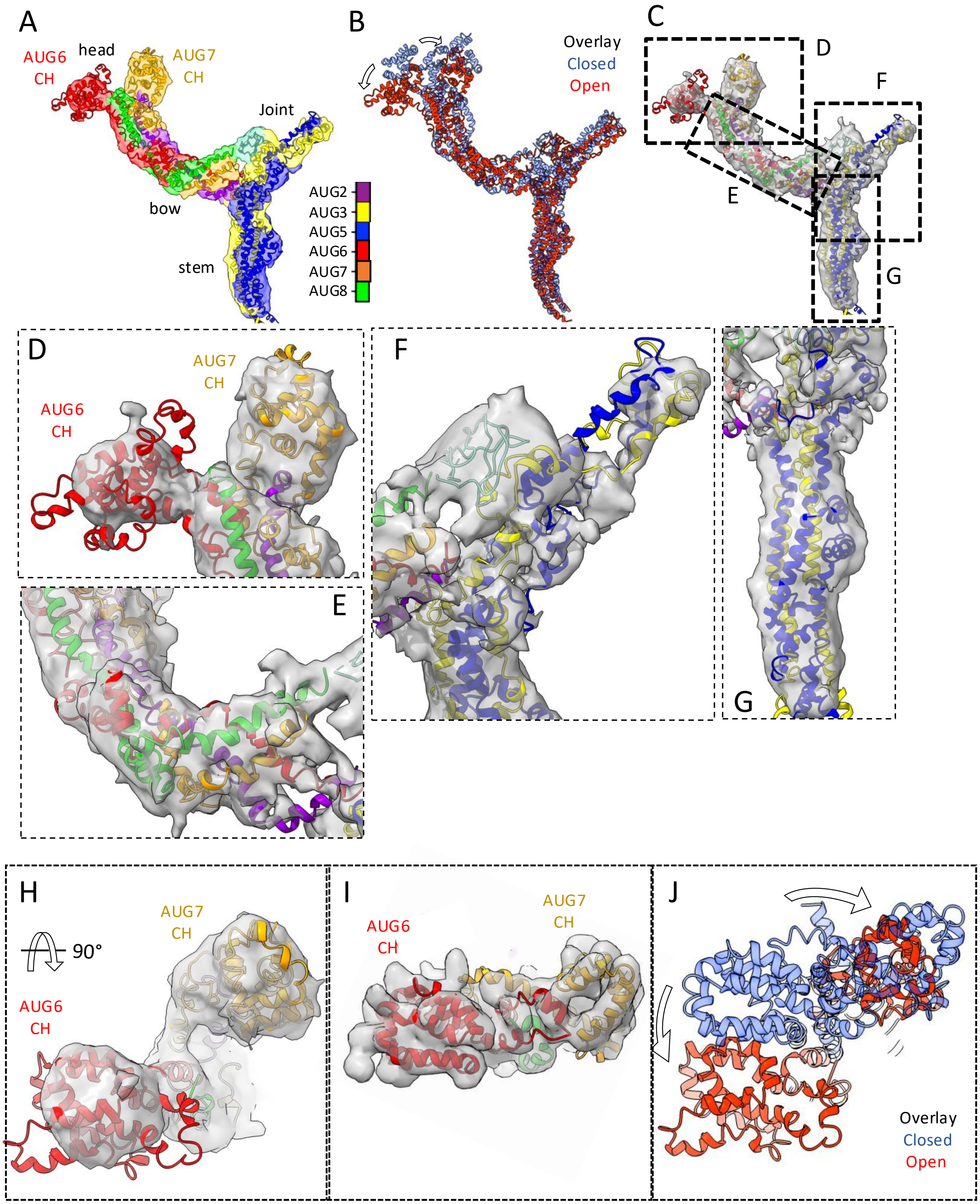

Supplementary Fig. 6: **Map to model view for the Augmin V-junction-stem in AUG6,7 CH dimer open state**

A) Segmented map fitted with modeled AUG1,2,3,4,5,7,8 regions into the V-junction-stem map (top) with the head region AUG6,7 CH-domain dimer in the open state. Subunit color guide shown in on right.
B) Overlay of the open state V-junction and closed state V-junction as shown in Figure 2B
C) A Guide to close up views of the modeled regions shown in A. The V-junction and stem map zones marked with D-I boxes.

D-G) Close-up views for different regions of V-junction stem model to map fits with guide to subunit colors shown in A.

H) Top end view of the head domain AUG6,7 CH-domain dimer in the open state

I) Top end view of the head domain AUG6,7 CH-domain dimer in the closed state

J) Top end view of the overlay of the ch-AUG6,7 dimer in the open and closed state shown in red and blue, respectively, showing the conformational transition of the CH-domains, suggesting AUG7 CH-domain moves more extensively than AUG6 Ch in the opening transition.

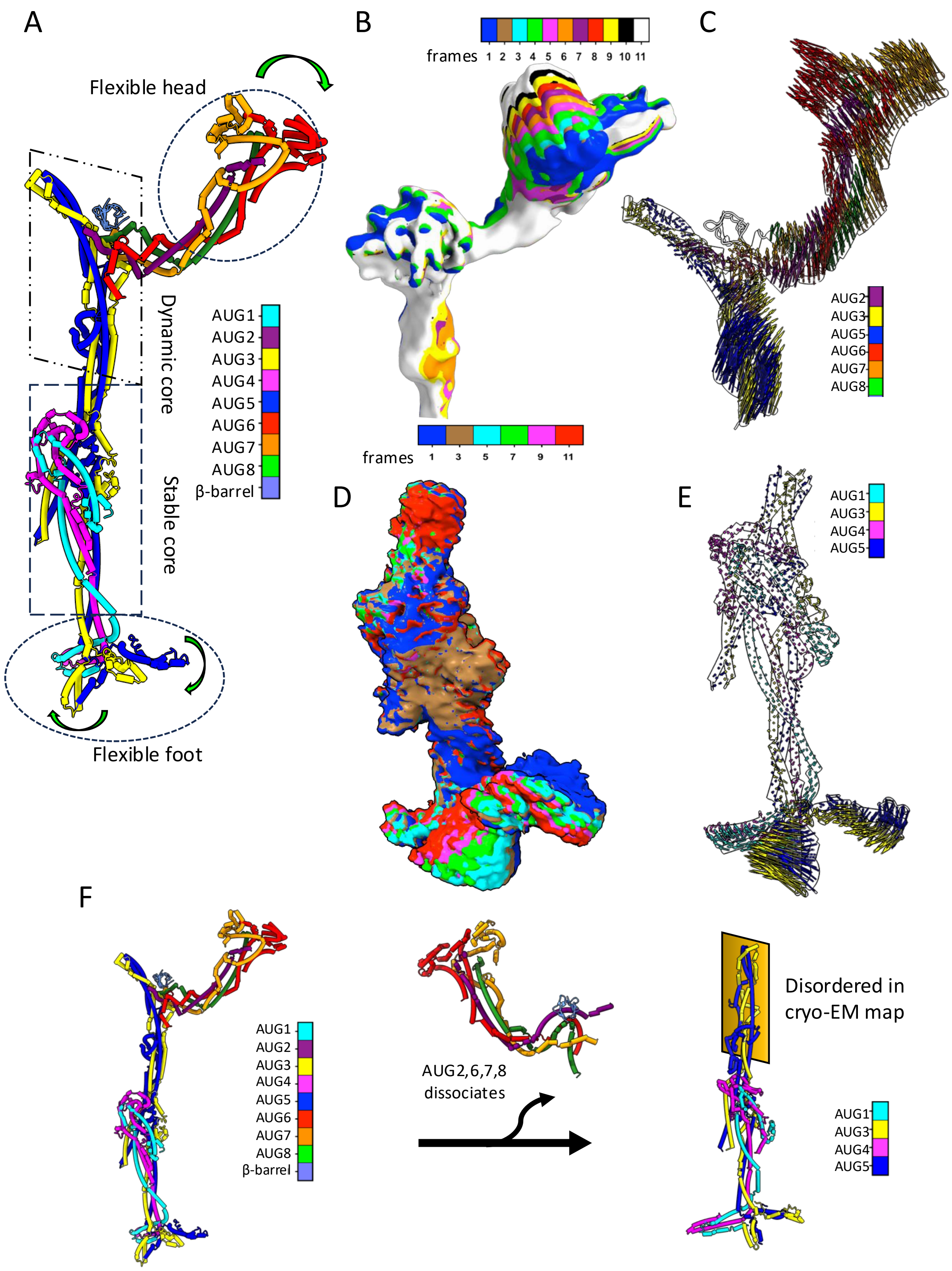

Supplementary Fig. 7: **Regional Flexibilities observed in Augmin structural analysis.**

A) Tube cartoon model for the Augmin hetero octamer showing the flexible regions in the assembly. The V-junction head domain and bow region show flexibility and the tripod region at the other end shows flexibility:
B) Low resolution frame maps for the V-junction stem region, generated in the 3D-Flex processing that marked with colors as shown in the guideline. These maps differ in the movement rotation of the V-junction bow and the head regions
C) Vectorial movement model showing the regions of the V-junction and stem that undergo movement. The magnitude of these movements is demonstrated by longer vector lines. The subunit color guide describes the subunits presented in the region.
D) Low resolution frame maps for the extended region, generated in the 3DFlex processing that marked with colors as shown in the guideline. These maps differ in the movement rotation of the leg region and the connected tripod region at the lowest part of the map.
E) Vectorial movement model showing the regions of the extended region map that undergo movement. The magnitude of these movements is demonstrated by size of the vector lines. The subunit color guide describes the subunits presented in the region.
F) A model describing the impact of the dissociation of AUG2,6,7,8 subcomplex leading to the destabilization of the structural stability of the AUG3,5 foldback zone in the extended region structure represented by AUG1,3,4,5.

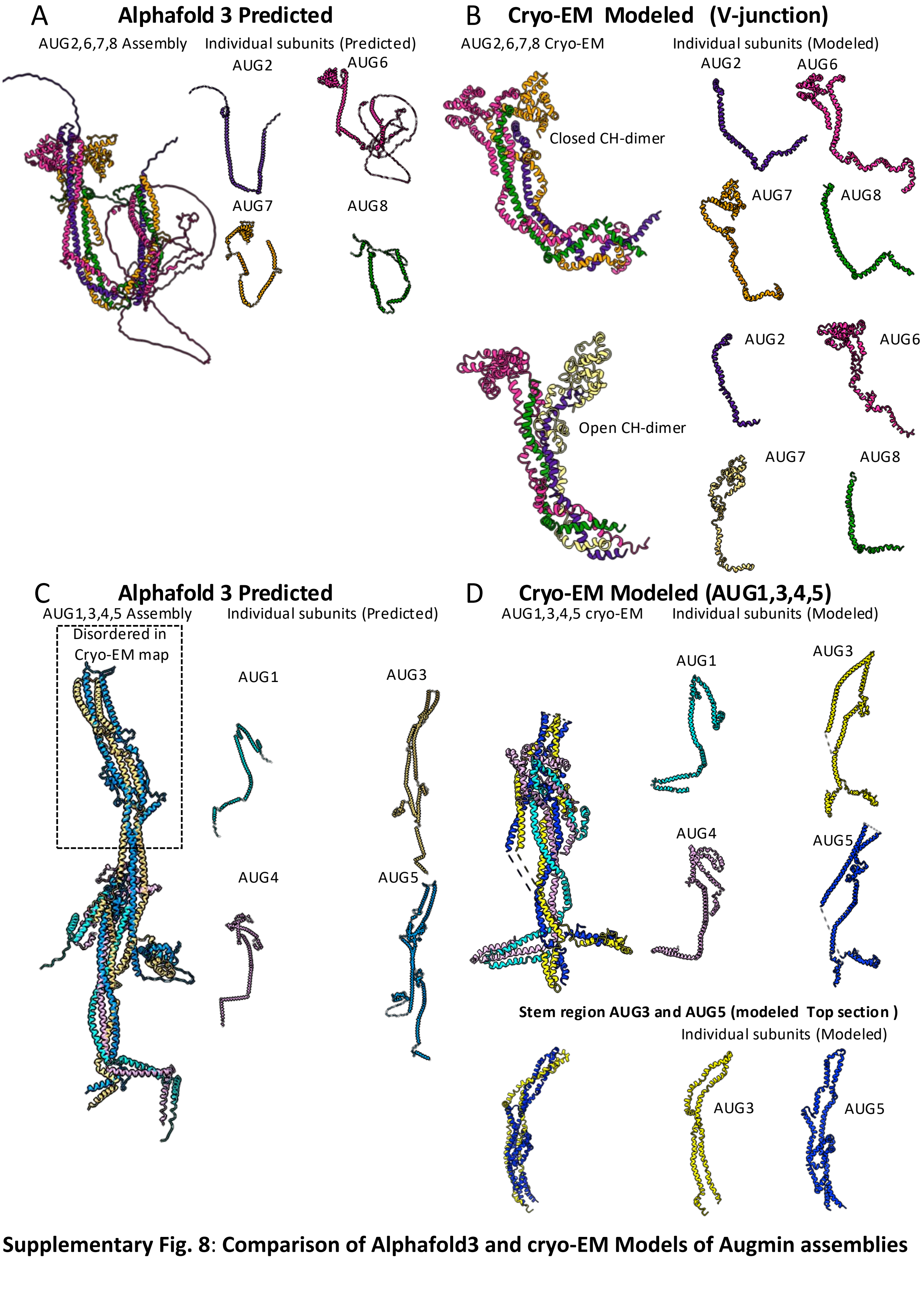

Supplementary Fig. 8: **Comparison of Alphafold3 and cryo-EM Models of Augmin assemblies**

and isolated subunits.

A) Left, AlphaFold 2 ribbon model for the *At* AUG2,6,7,8 assembly: right model for isolated AUG2,6,7,8 subunits.

B) Top left, Cryo-EM ribbon model for the *At* AUG2,6,7,8 assembly in the CH-dimer closed state; top right model for isolated AUG2,6,7,8 subunits. Bottom left, Cryo-EM ribbon model for the *At* AUG2,6,7,8 assembly in the CH-dimer open state; bottom right model for isolated AUG2,6,7,8 subunits.

C) Left, AlphaFold 2 ribbon model for the *At* AUG1,3,4,5 assembly with a box marking the AUG3,5-foldback zone, which is not observed in the AUG1,3,4,5 cryo-EM map of the extended region: right, models for isolated AUG1,3,4,5 subunits. Note the open shape of the end of the extended domain.

D) Left, Cryo-EM ribbon model for the *At* AUG1,3,4,5 assembly of the extended region; Top right, models for isolated AUG1,3,4,5 subunits in the AUG,1,3,4,5 extended region map. Bottom right, models for the AUG3, AUG5 and their assembly in the foldback zone observed in the AUG1,3,4,5,6,7,8 map of the V-junction and stem.

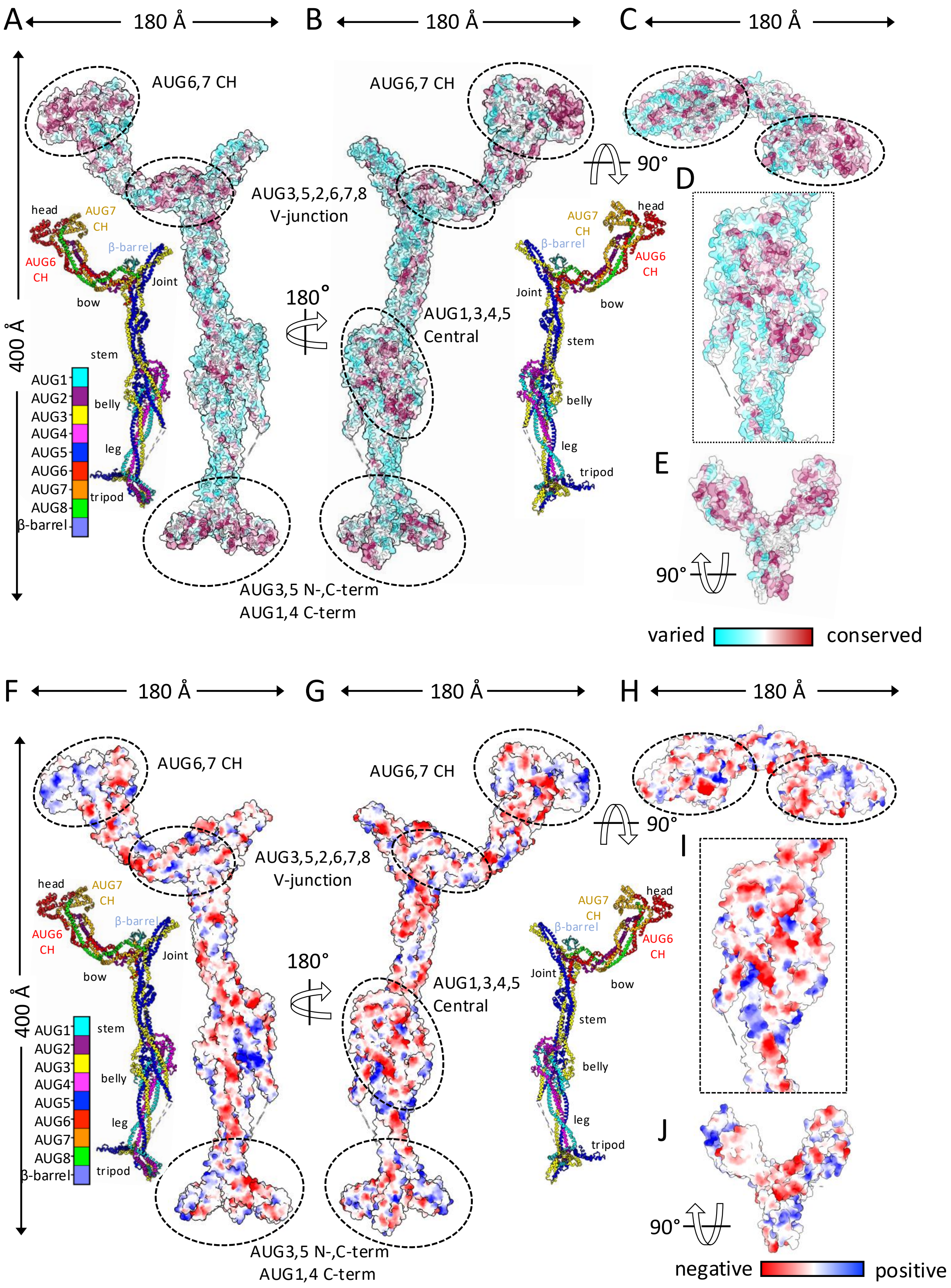

Supplementary Fig. 9: **Charge distribution and Sequence Conservation mapped on Augmin hetero-octamer (AUG1,2,3,4,5,6,7,8) model**.

A-C) A and B show two side views of the sequence conservation plotted by color on the Augmin surface model. The model is rendered using the color scheme shown on the top right. Inset models shown. A and B show the two inset side views of the Augmin ribbon model in the same views to help orient the view to the structure. C, Top shows the top end view of the V-junction. Middle shows close up view on the belly region shown in B. Bottom shows the view from the below the Tripod region. The surface conservation of the Augmin assembly structure suggests regions of critical functional importance in the Augmin structure which are labeled by ellipses.

F-J) F and G show two side views of the surface charge distribution plotted by color on the Augmin surface model. The model is rendered using the color scheme shown on the top right. Inset models shown. F and G show the inset two side views of the Augmin ribbon model in the same views to help orient the view to the structure. H, Top shows the top end view of the V-junction. I, shows close up view on the belly region shown in F. J, shows the view from the below the Tripod region. The charge distribution marks the four critical regions of importance in the Augmin structure which are labeled by ellipses.

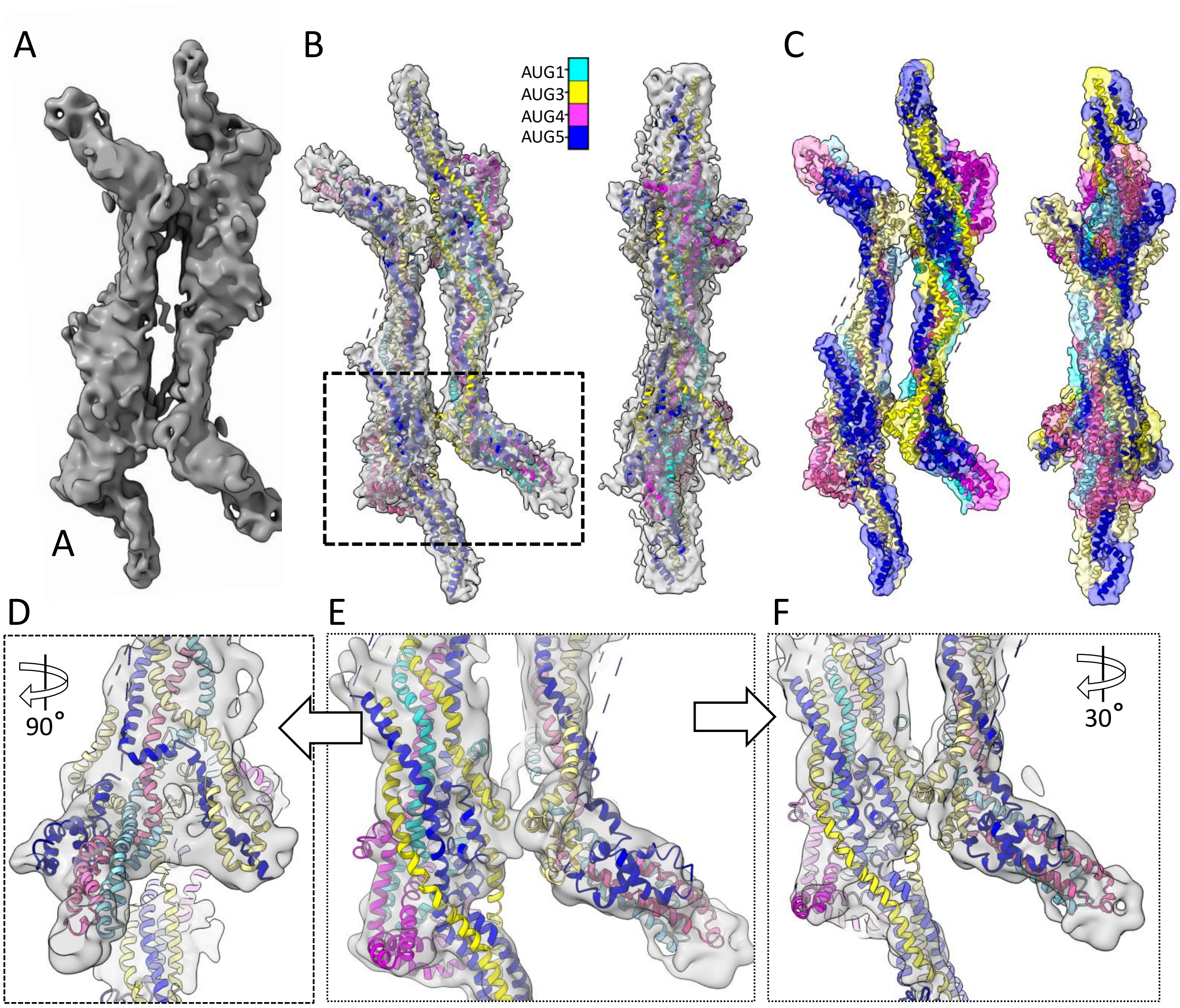

Supplementary Fig. 10: **Details of the Augmin dimer cryo-EM map to model**

A) The AUG1,2,3,4,5,6,7,8 Augmin C2 dimer map generated as described in Supplementary Fig. 2

B) Two views of the assemblies of AUG1,3,4,5 built into the raw cryo-EM map (grey).

C) Two views of the assemblies of AUG1,3,4,5 built presented into the segmented cryo-EM map (multi-color). Subunits and segments are presented in the colors described in the palette above.

D-F) three rotated views of the Augmin Dimer map interfaces between the folded tripod region and the belly region. The map is shown in raw grey color while each subunit is presented in ribbon format following the color pallet

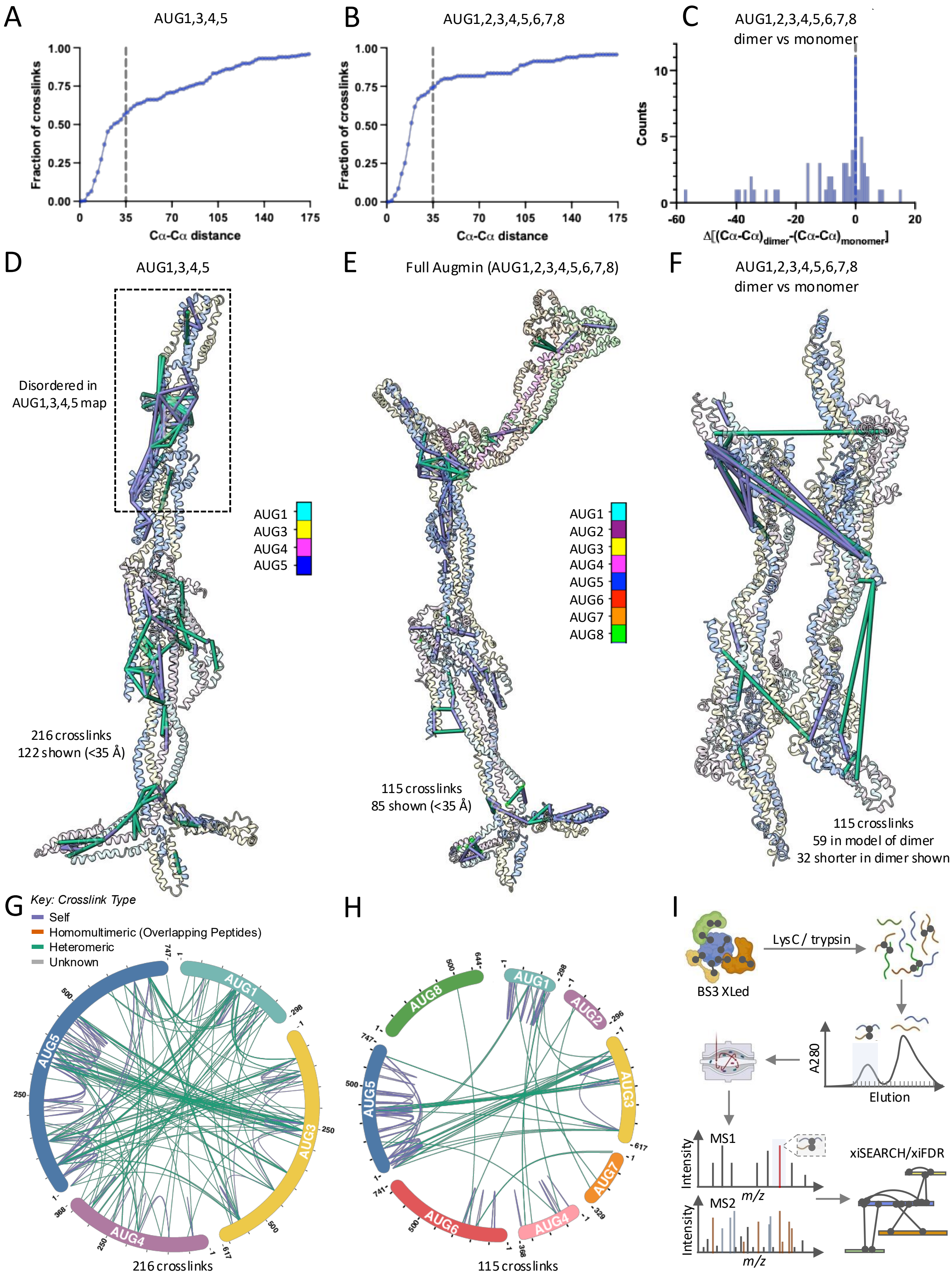

Supplementary Fig. 11: Crosslinking Mass spectrometry (XLMS) of AUG1,3,4,5 and

AUG1,2,3,4,5,6,7,8

A) CDF plot for hetero-tetrameric (AUG1,3,4,5) Augmin showing the fraction of the 216 crosslinks whose Cα-Cα distance is less than the indicated distance. The line denotes the 35 Å cutoff.
B) CDF plot for hetero-octameric (AUG1,2,3,4,5,6,7,8) Augmin showing the fraction of the 115 crosslinks whose Cα-Cα distance is less than the indicated distance. The line denotes the 35 Å cutoff.
C) Histogram showing the difference in the Cα-Cα distance between residue pairs in the context of the antiparallel dimer structural model relative to the monomer structural model. Only 59 crosslinks that can be mapped in the antiparallel dimer model are shown.
D) Crosslinks identified in AUG1,3,4,5, illustrated in the context of the cryo-EM structural model of AUG1,3,4,5. In total, 216 unique residue pairs were identified, of which the 122 with Cα-Cα distances less than 35 Å are displayed.
E) Crosslinks identified in AUG1,2,3,4,5,6,7,8 illustrated in the context of the cryo-EM structural model of AUG1,2,3,4,5,6,7,8. In total, 115 unique residue pairs were identified, of which the 85 with Cα-Cα distances less than 35 Å are displayed.
F) Crosslinks identified in the AUG1,2,3,4,5,6,7,8 illustrated in the context of the cryo-EM structural model of the anti-parallel Augmin dimer. The 32 crosslinks which have shorter Cα-Cα distances in the context of the anti-parallel dimer compared to the monomer are shown.
G) Connectogram showing all 216 crosslinks identified in AUG1,3,4,5.
H) Connectogram showing all 115 crosslinks identified in AUG1,2,3,4,5,6,7,8.
I) Experimental scheme for XLMS

A Protein interaction network of AUG1,3,4,5 and contaminating proteins

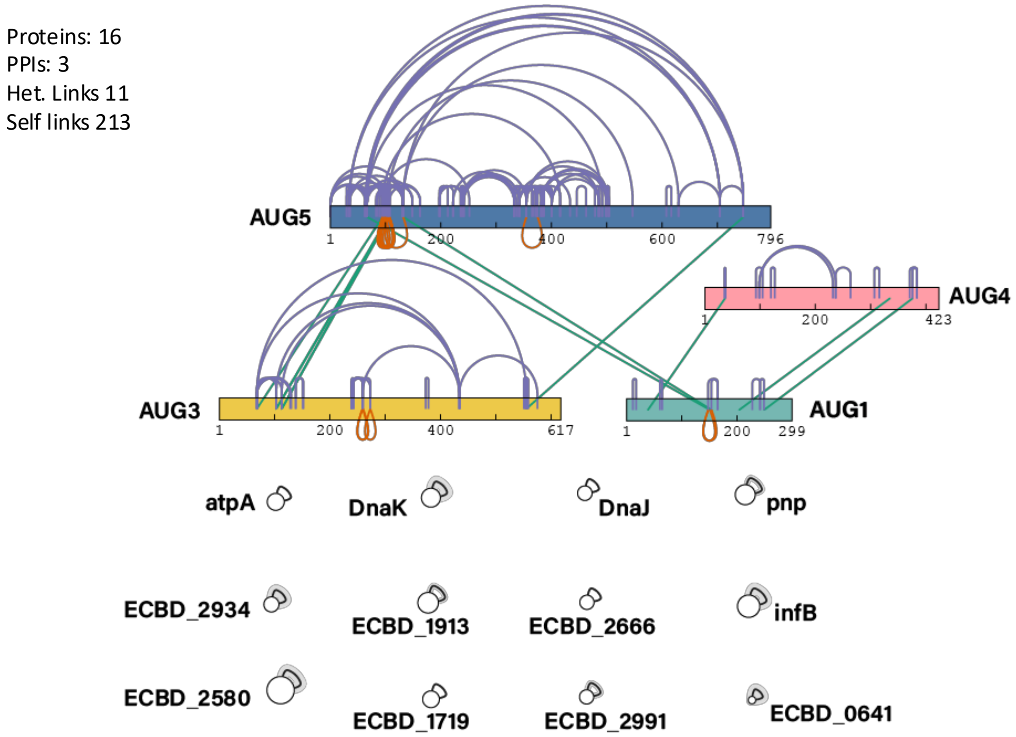

B Protein interaction network of AUG1,2,3,4,5,6,7,8 and contaminating proteins

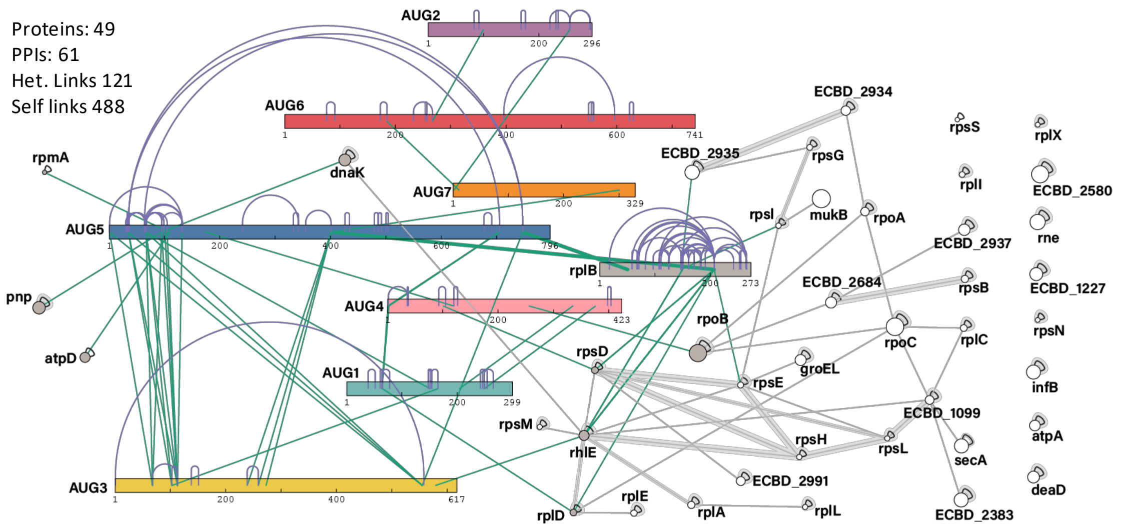

Supplementary Fig. 12: Extended XL-MS searches including contaminants

A) Connectogram showing crosslinks identified between AUG1,3,4,5 and 46 top contaminant proteins from *E. coli.* No direct crosslink between Augmin hetero-tetramer and an *E. coli* protein is detected.

B) Connectogram showing crosslinks identified between AUG1,2,3,4,5,6,7,8 and 42 top contaminant proteins from *E. coli.* Crosslinks between Augmin hetero-octamer and nine *E. coli* proteins are detected, including RplB.

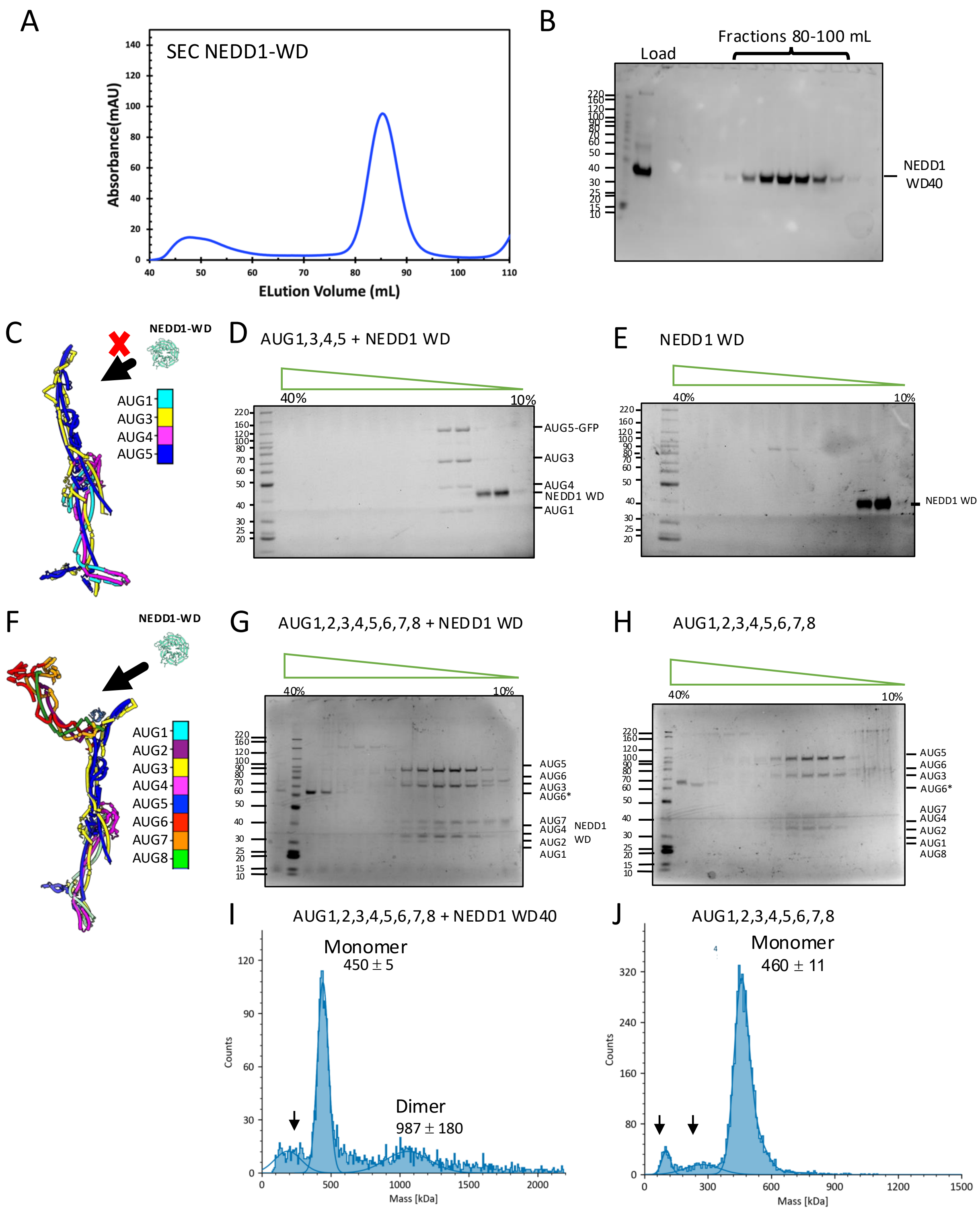

Supplementary Fig. 13: Purification of *At* NEDD1 WD-β-propeller and biochemical reconstitution of NEDD1-WD-β-propeller with AUG1,3,4,5 and AUG1,2,3,4,5,6,7,8.

A) SEC purification chromatogram of insect cell expressed At NEDD1-WD β-propeller using Superdex 200
B) SDS-PAGE of SEC-purified NEDD1-WD fractions revealing the solubility and monodisperse behavior of the protein as globular entity.
C) Model for NEDD1 WD β-propeller not binding to Augmin hetero-tetramer (AUG1,3,4,5)
D) 10-40% Sucrose density gradient of AUG1,3,4,5 with NEDD1-WD showing the isolated and non-overlapping and separate migration of these two entities
E) 10-40% Sucrose density gradient of NEDD1-WD showing its matching migration of pattern alone with panel C.
F) Model for NEDD1 WD β-propeller binding to Augmin hetero-octamer (AUG1,2,3,4,5,6,7,8)
G) 10-40% Sucrose density gradient of AUG1,2,3,4,5,6,7,8 with NEDD1-WD showing they co-elute together as a collection of proteins with fractions showing their overlay or migration.
H) 10-40% Sucrose density gradient of AUG1,2,3,4,5,6,7,8 showing the subunits co-elute forming a single entity with co-migration of all relevant subunits.
I) Mass photometry results (N=3) showing monomer and dimer population in presence of NEDD1-WD. Arrow indicate the broken-down complex of unknown origin.
J) Mass photometry results (N=3) showing mostly monomer population in absence of NEDD1-WD. Arrows indicate the broken-down complex of unknown origin.

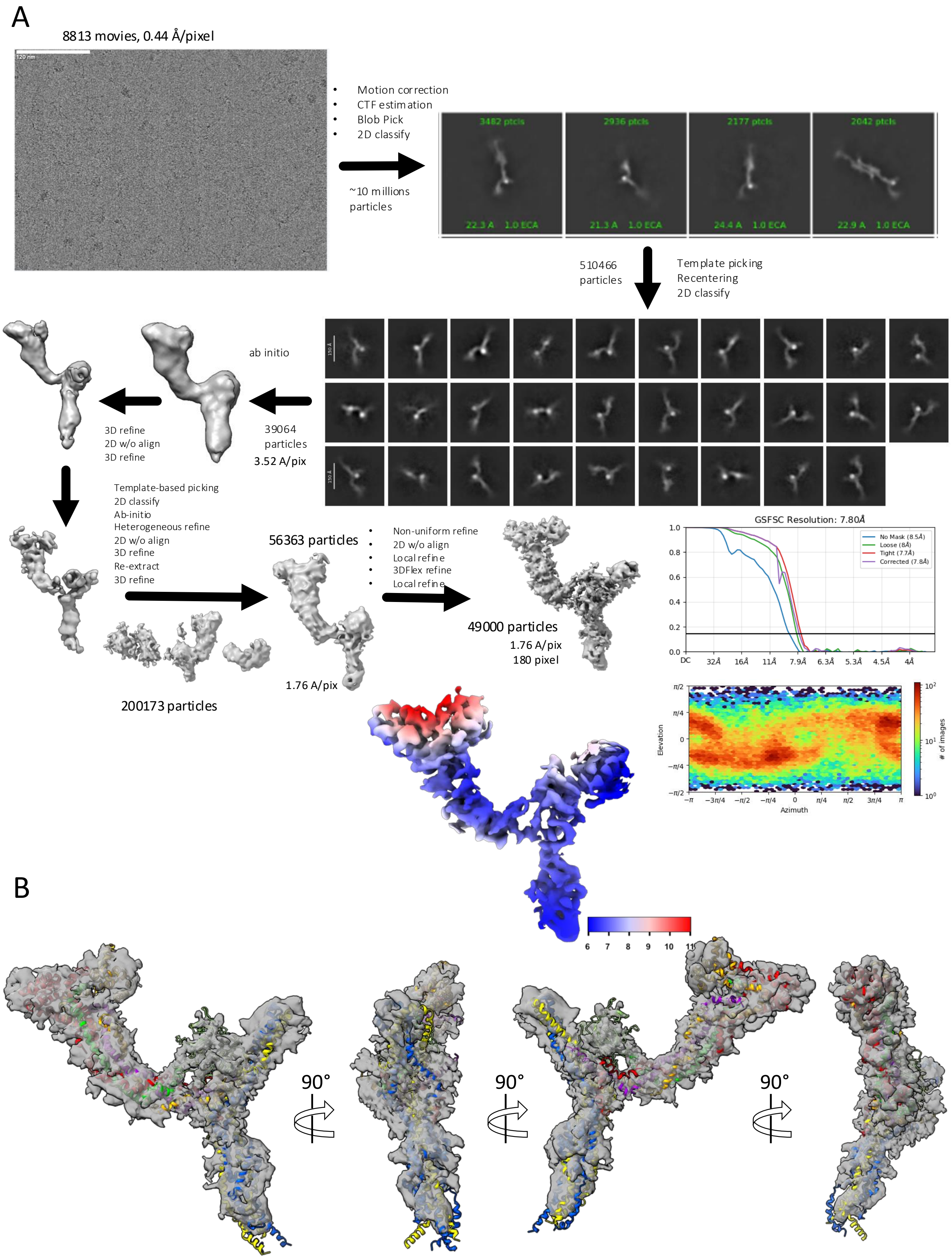

Supplementary Fig. 14: Cryo-EM structure determination of the NEDD1 β-propeller-Augmin-V-junction and stem region.

A) From top to bottom: Cryo-EM data for AUG1,2,3,4,5,6,7,8 +NEDD1 WD assemblies (representative image shown on top left) were pre-processed then picked and 2D-classified with many rounds of 2D-classification leading a mixture of full monomeric or dimeric assemblies Template picking and recentering and 2D-classification led improved 2D-classes of V-junction-stem regions of Augmin. We note the density of the signal, likely representing the NEDD1-WD binding site, at the top of the V-junction is higher than Augmin -V-junction top section alone. These 2D-class average images were then to generate multi *ab initio* model which was 3D-auto-refined and classified leading to a 10 Å resolution structure.

B) Top, Fourier Shell correlation (FSC) for the final NEDD1-wd bound AUG1,2,3,4,5,6,7,8 V-junction stem. Middle, the angular distribution of the resulting map. Bottom,

C) Model comparison of the NEDD1-WD-AUG1,3,4,5 V-junction-stem region map to a model generated for the complex showing the close overlay in many orientations, particularly the large size of the density representing NEDD1 compared density in AUG1,2,3,4,5,6,7,8 which is bound by the weaker β-barrel containing densities.

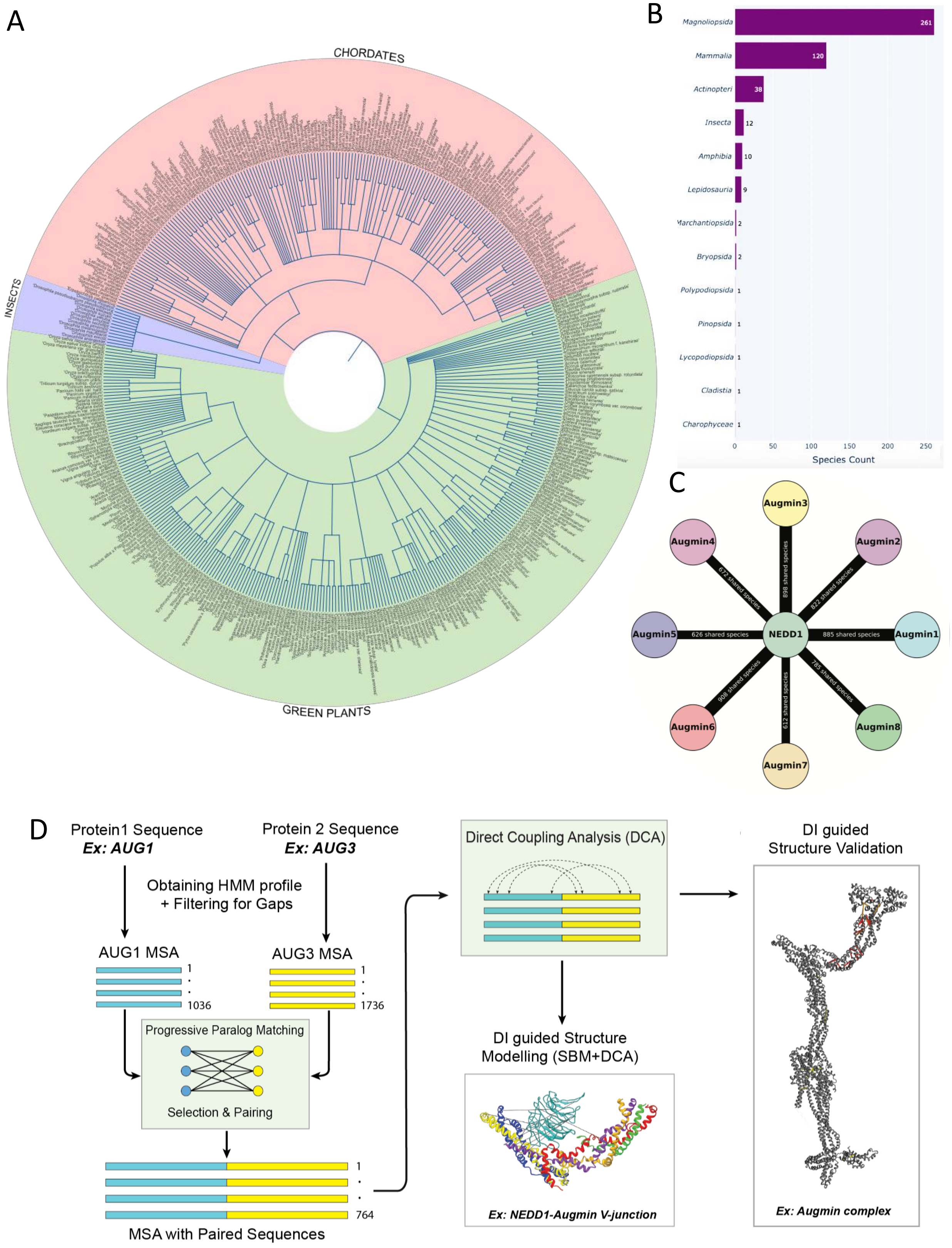

Supplementary Fig. 15: Coevolutionary analysis of the Augmin hetero-octamer and its interaction with NEDD1.

A) Evolutionary tree of species containing all eight Augmin subunits and NEDD1, spanning plants, chordates and insects.
B) Bar chart of species counts per class possessing the complete set of eight Augmin subunits and NEDD1.
C) Species overlap between orthologs of NEDD1 and each of the Augmin subunits.
D) Workflow for coevolutionary analysis using direct coupling analysis (DCA): left—MSA construction and filtering; lower left—application of progressive paralog matching (PPM) for generation of paired MSAs; top center—DCA inference of DI pairs from paired MSAs; lower center—DI guided structural modelling of NEDD1-Augmin V-junction; right—DI guided structural validation of Augmin hetero-octamer.

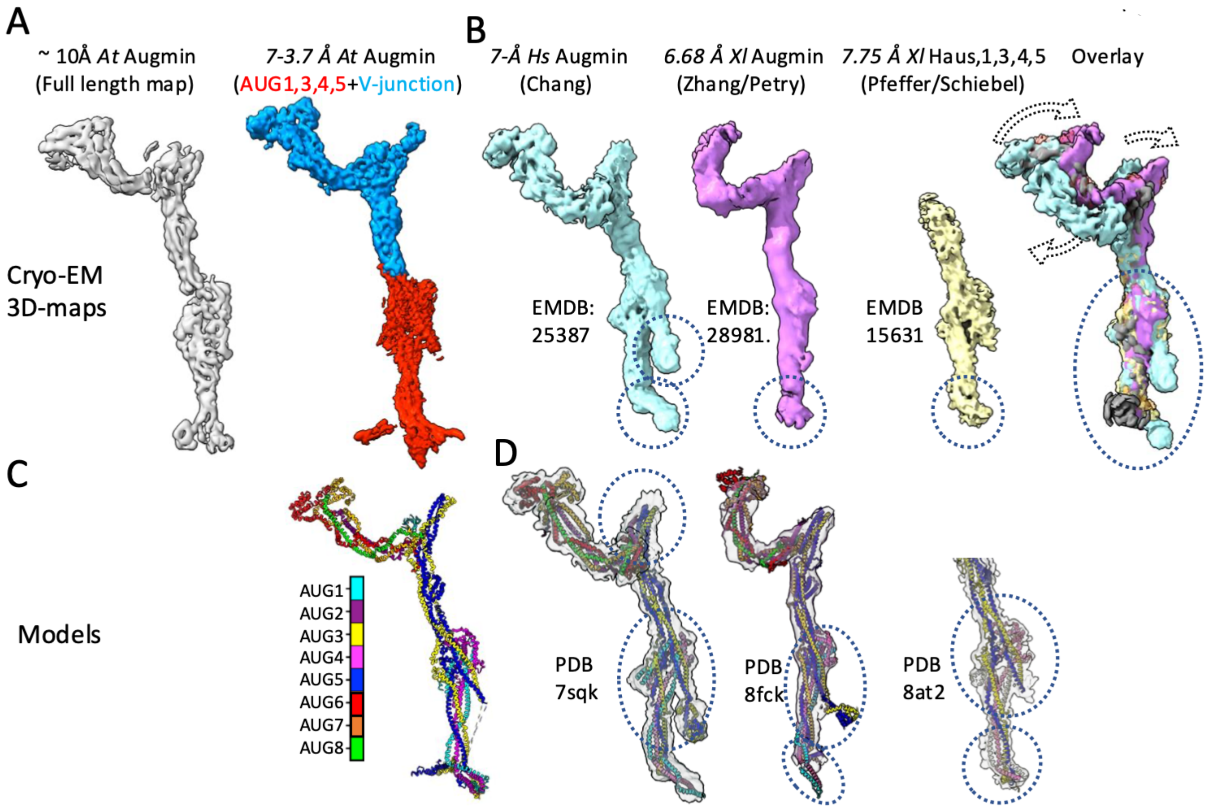

Supplementary Fig. 16: comparison of our plant Augmin structures to previously published Augmin structures

A) Left, full Augmin assembly map generated as described in Figure 2; right the overlay of the full Augmin assembly map onto the 7.3 Å Augmin V-junction-stem map (blue) and 3.7 Å extended region domain map (red).

B) Left, the cryo-EM map for full Augmin generated Gabel *et al* (cyan). Second from left, the cryo-EM map for full Augmin generated by Travis *et al* (purple). Third left, cryo-EM map of the Haus1,3,4,5 region generated by Zuppa *et al* (purple)[1-3].

C) *De novo* At Augmin assembly presented in this manuscript

D) Left, Full model fit into the Cryo-EM density map placed into the *Gabel et al* (pink); second from left, Travis *et al*. Right, Zuppa *et al* and colleagues model fit into the cryo-EM of Haus 1,3,4,5 assemblies showing its nearly matching the shape and size of the AUG1,3,4,5 structure presented here[1-3].

Supplementary table 1: AUG1,2,3,4,5,6,7,8 pairing for DCA analyses

S.No	Chain pairs	Sequence length	Sequences in paired MSA
1	A-C	559	764
2	A-D	534	637
3	A-E	942	594
4	B-C	451	671
5	B-E	834	597
6	B-F	424	681
7	B-G	509	295
8	B-H	493	438
9	C-D	495	667
10	C-E	903	612
11	D-E	878	600
12	F-C	493	889
13	F-E	876	624
14	F-G	551	287
15	F-H	235	492
16	G-C	578	275
17	G-E	961	270
18	G-H	620	316
19	H-C	562	466
20	H-E	945	369

Supplementary table 2: Lengths and MSA sequence totals for AUG and NEDD1 subunits

Protein	Chain ID	UniProt ID	Sequence length	Total sequences in MSA	Total sequences in filtered MSA
AUG 1	A	F4IK01	299	1500	1036
AUG 2	B	O48767	296	1621	912
AUG 3	C	Q0WQE7	617	2109	1736
AUG 4	D	Q8GYM3	423	1509	972
AUG 5	E	Q9FMB4	796	1582	812
AUG 6	F	Q94BP7	741	2986	2223
AUG 7	G	Q0WTP1	329	665	350
AUG 8	H	Q9SUH5	644	3608	2503
NEDD1	I	B3H5K9	310	4500	3500

Supplementary table 3: DCA DI pairs in AUG1,2,3,4,5,6,7,8 subunits

Serial No.	Chain 1	Residue 1	Chain 2	Residue 2	p-value*
**1**	A	128	C	139	0.11
**2**	A	146	C	120	0.11
**3**	A	124	E	583	0.16
**4**	A	139	E	619	0.16
**5**	A	83	E	589	0.16
**6**	A	82	E	622	0.16
**7**	B	45	G	151	0.09
**8**	B	91	G	202	0.09
**9**	B	43	G	152	0.09
**10**	B	60	G	190	0.09
**11**	B	50	G	107	0.09
**12**	B	36	G	120	0.09
**13**	B	91	G	209	0.09
**14**	B	29	G	106	0.09
**15**	B	108	G	213	0.09
**16**	B	114	G	236	0.09
**17**	B	62	G	190	0.04
**18**	B	54	G	159	0.04
**19**	B	59	G	155	0.04
**20**	B	51	G	159	0.04
**21**	B	44	G	151	0.002
**22**	B	47	G	151	0.002
**23**	B	43	G	151	0.002
**24**	B	50	G	155	0.002
**25**	B	60	G	188	0.002
**26**	B	44	G	148	0.002
**27**	C	65	E	68	0.11
**28**	C	201	E	514	0.11
**29**	C	194	E	525	0.11
**30**	C	204	E	528	0.11
**31**	C	52	E	59	0.11
**32**	C	235	E	367	0.11
**33**	G	245	H	163	0.02
**34**	G	142	H	103	0.02
**35**	G	245	H	162	0.02
**36**	G	206	H	139	0.02
**37**	G	232	H	163	0.02
**38**	G	225	H	150	0.02
**39**	G	225	H	149	0.01
**40**	G	206	H	133	0.01
**41**	G	198	H	132	0.01
**42**	G	111	H	72	0.02

Supplementary table 4: DCA MSA sequence DI pairing NEDD1- with AUG2,3,5,6,7,8

S.No	Chain pairs	Sequence length	Total sequences in paired MSA
1	NEDD1-B	501	708
2	NEDD1-C	570	1009
3	NEDD1-E	953	630
4	NEDD1-F	543	1145
5	NEDD1-G	628	308
6	NEDD1-H	612	533

Supplementary table 5: NEDD1 WD- AUG2,5,6 DCA DI pairs

Pair No	NEDD1 Residue	NEDD1 Chain	Interact subunit residue	Interact Subunit chain
**1**	106	I	759	F
**2**	106	I	756	F
**7**	288	I	622	E
**9**	47	I	387	B
**10**	47	I	397	B

Supplementary References

1 Gabel, C. A. *et al.* Molecular architecture of the augmin complex. *Nat Commun* **13**, 5449 (2022). https://doi.org:10.1038/s41467-022-33227-7

2 Travis, S. M. *et al.* Integrated model of the vertebrate augmin complex. *Nat Commun* **14**, 2072 (2023). https://doi.org:10.1038/s41467-023-37519-4

3 Zupa, E. *et al.* The augmin complex architecture reveals structural insights into microtubule branching. *Nat Commun* **13**, 5635 (2022). https://doi.org:10.1038/s41467-022-33228-6
