# Supplementary material for: Cryo-EM structures of plant Augmin reveal coiled-coil assembly, antiparallel dimerization, and NEDD1 binding
Source: Nat Commun. 2025 Dec 12;16:11440. doi: 10.1038/s41467-025-66332-4 (PMC12748588; doi:10.1038/s41467-025-66332-4)
Supplement: Supplementary file 2 — Description of Additional Supplementary Files [file 41467_2025_66332_MOESM2_ESM.pdf]

Description of Additional Supplementary Files

File Name: Supplementary Data 1
Description: List of all unique crosslinked peptides for AUG1,3,4,5 and AUG1,2,3,4,5,6,7,8 identified with either a restricted search or expanded search including contaminants

File Name: Supplementary Movie 1
Description: Summary of Cryo-EM structures, segmented maps and models of plant Augmin
The video shows the hetero-octameric (AUG1,2,3,4,5,6,7,7,8) Augmin full length 12-Å cryo-EM map, followed by the placement of the raw 7.3-Å V-junction stem AUG1,2,3,4,5,6,7,8 cryo-EM structure (blue) and 3.7-Å AUG1,3,4,5 cryo-EM structure of the extended region of Augmin (red) into the 10Å full Augmin map. The segmented maps and fitted models are shown for 7.3 Å V-junction stem in the closed state, 3.7Å extended region, 10-Å V-junction stem in the open state, and 12-Å structure of the NEDD1-WD-β-propeller bound V-junction stem. The overlap 20 Å zones between the extended region and the V-junction stem maps are marked for making composite *de novo* models. The model of the full hetero-octameric (AUG,1,2,3,4,5,6,7,8) Augmin in the closed state, then open state, and the NEDD1-WD-β-propeller bound states are shown in succession. Finally, the three full Augmin models are overlaid with Augmin closed state (blue), Augmin open sate (green) and Augmin-NEDD1-WD-β-propeller bound state (red) are all compared showing the nature of the V-junction transitions.
